# CircMiMi: a stand-alone software for constructing circular RNA-microRNA-mRNA interactions across species

**DOI:** 10.1186/s12859-022-04692-0

**Published:** 2022-05-06

**Authors:** Tai-Wei Chiang, Te-Lun Mai, Trees-Juen Chuang

**Affiliations:** 1 Genomics Research Center, Academia Sinica, Taipei, Taiwan; 2 Department of Life Science, National Taiwan University, Taipei, Taiwan

**Keywords:** Circular RNA, microRNA, Regulatory interaction, Alignment ambiguity, Reverse complementary sequence, Autism spectrum disorder

## Abstract

**Background:**

Circular RNAs (circRNAs) are a class of non-coding RNAs formed by pre-mRNA back-splicing, which are widely expressed in animal/plant cells and often play an important role in regulating microRNA (miRNA) activities. While numerous databases have collected a large amount of predicted circRNA candidates and provided the corresponding circRNA-regulated interactions, a stand-alone package for constructing circRNA-miRNA-mRNA interactions based on user-identified circRNAs across species is lacking.

**Results:**

We present CircMiMi (**circ**RNA-**mi**RNA-**m**RNA **i**nteractions), a modular, Python-based software to identify circRNA-miRNA-mRNA interactions across 18 species (including 16 animals and 2 plants) with the given coordinates of circRNA junctions. The CircMiMi-constructed circRNA-miRNA-mRNA interactions are derived from circRNA-miRNA and miRNA-mRNA axes with the support of computational predictions and/or experimental data. CircMiMi also allows users to examine alignment ambiguity of back-splice junctions for checking circRNA reliability and examine reverse complementary sequences residing in the sequences flanking the circularized exons for investigating circRNA formation. We further employ CircMiMi to identify circRNA-miRNA-mRNA interactions based on the circRNAs collected in NeuroCirc, a large-scale database of circRNAs in the human brain. We construct circRNA-miRNA-mRNA interactions comprising differentially expressed circRNAs, and miRNAs in autism spectrum disorder (ASD) and cross-species analyze the relevance of the targets to ASD. We thus provide a rich set of ASD-associated circRNA-miRNA-mRNA axes and a useful starting point for investigation of regulatory mechanisms in ASD pathophysiology.

**Conclusions:**

CircMiMi allows users to identify circRNA-mediated interactions in multiple species, shedding light on regulatory roles of circRNAs. The software package and web interface are freely available at https://github.com/TreesLab/CircMiMi and http://circmimi.genomics.sinica.edu.tw/, respectively.

**Supplementary Information:**

The online version contains supplementary material available at 10.1186/s12859-022-04692-0.

## Background

Circular RNAs (circRNAs) are a class of long non-coding RNAs produced by pre-mRNA back-splicing with a distinct single-strand, non-polyadenylated circular loop [1]. They were observed to be more stably expressed than their corresponding co-linear mRNA isoforms [2–4]. Genome-wide analyses of high-throughput RNA sequencing (RNA-seq) revealed that circRNAs were abundant in animals [3, 5, 6] and plants [7]. Some circRNAs are evolutionarily conserved in terms of both circle sequence and expression across mammals [5, 8, 9]. The best understood function of circRNAs is the regulatory role in regulating microRNA (miRNA) activities, with either miRNA sponges or scaffolds [10]. Accumulating evidence shows that circRNA-miRNA regulatory axes can involve in cancer-related [11] and neurobiological pathways [12, 13], suggesting the potential implications of circRNA-miRNA-mRNA regulatory pathways in pathophysiology of human diseases.

Nowadays, numerous tools [14] and databases [15] have been developed for identification and analysis of circRNAs, providing a large amount of publicly accessible circRNA resources. However, there are great discrepancies among the circRNA candidates identified by different circRNA detectors, implying the uncertainty of detected circRNAs [16, 17]. Indeed, a considerable number of circRNAs detected by many currently-available tools were still derived from ambiguous alignments with an alternative co-linear explanation or multiple hits [18, 19]. It is worthwhile to reexamine the alignment ambiguity of the circRNAs for further analyses. In terms of circularization, previous studies demonstrated that back-splicing can be promoted by reverse complementary sequences (RCSs) residing in the introns flanking the circularized exons [3, 4, 20] and affected by the competition of RCSs across flanking introns (RCS_across_) or within individual flanking introns (RCS_within_) [20]. Genome-wide analyses of circRNA-flanking introns further revealed that the number of RCS_across_ was generally larger than that of RCS_within_ [4, 20], suggesting the association between RCSs and circularization. It is helpful to examine the existence of RCSs for further investigation of circRNA formation.

While several circRNA databases or web-based tools [14, 15, 21] also provide predictions of circRNA-miRNA interactions, they are often hampered by one or more of the following limitations: (1) the provided circRNA-regulated axes are based on the circRNA candidates identified/collected by the known circRNA databases only; (2) the examined circRNAs focus on human or limited species; (3) the number of query circRNAs is limited; or (4) the corresponding circRNA-miRNA or miRNA-mRNA axes are derived from computational predictions only. To address all the above limitations, we present CircMiMi (**circ**RNA-**mi**RNA-**m**RNA **i**nteractions), a Python-based software, to identify circRNA-miRNA-mRNA interactions across 18 species (including 16 animals and 2 plants) according to user-provided coordinates of circRNA junctions. It is noteworthy that the CircMiMi-identified circRNA-miRNA-mRNA interactions are derived from circRNA-miRNA and miRNA-mRNA axes with the support of computational predictions and/or experimental data (e.g., CLIP or microarray data). The executable files for visualizing the constructed circRNA-miRNA-mRNA regulatory axes are provided. CircMiMi also provides optional functions for examining alignment ambiguity of circRNAs and RCSs across/within flanking sequences of back-splice junctions (BSJs). We further utilize CircMiMi to construct circRNA-miRNA-mRNA interactions based on the circRNAs collected in NeuroCirc [22], which deposits more than 26,000 circRNAs derived from human brain tissues or neuronal cells. According to differentially expressed circRNAs (DE-circRNAs), miRNAs (DE-miRNAs) in autism spectrum disorder (ASD), a rich set of ASD-associated circRNA-miRNA-mRNA axes is also provided. With the ability in identifying circRNA-miRNA-mRNA axes across species, CircMiMi also identifies mouse circRNA-miRNA-mRNA axes based on human-mouse orthologous circRNAs. Enrichment analysis further shows that the targets of the DE-circRNA-associated axes are enriched for ASD risk genes. CircMiMi is highly automated and modularized, which is convenient to be expanded to include new experimental data in the future.

## Methods

### Alignment ambiguity and RCS checking

**Fig. 1:**
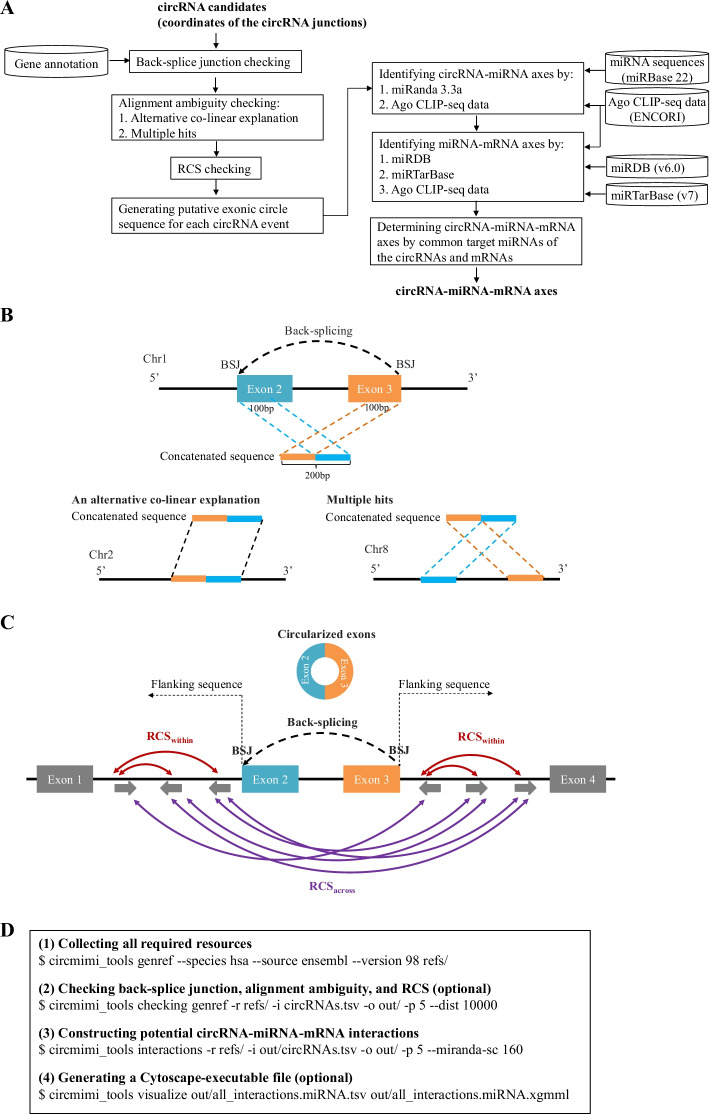
Overview of CircMiMi. **a** Flowchart of the overall pipeline. **b** Schematic illustration of back-splicing events arising from ambiguous alignments with an alternative co-linear explanation (left) and multiple hits (right). For the left panel, the concatenated sequence of the back-splicing event has an alternative co-linear explanation on another chromosome. For the right panel, the concatenated sequence also non-co-linearly maps to another genomic region. **c** Schematic illustration of sequences flanking circularized exons and the corresponding RCS_across_ and RCS_within_. In this case, RCS_across_ = 5, RCS_within_ = 4, and RCS_across_—RCS_within_ = 1. **d** The four main CircMiMi command lines: (1) collecting all required resources; (2) checking BSJ, alignment ambiguity, and RCS; (3) identifying circRNA-miRNA-mRNA interactions; and (4) generating a Cytoscape-executable file. For (2), “–dist” represents the considered length of the flanking sequences (± N nucleotides of the back-splice site; default value = 10,000). For (3), “–miranda-sc” represents the miRanda score threshold (default value = 155). For (2) and (3), “-p” represents the number of processor cores (default value = 1)

The workflow of CircMiMi is illustrated in Fig. 1a. CircMiMi can automatically collect the newest version of annotation information, if users do not specify the version. By inputting the coordinates of the BSJs, CircMiMi can automatically determine donor and acceptor sites of the circRNA candidates according to the input coordinates and strands. Since previous studies suggested that BSJs required canonical splice signals and tended to be located at well-annotated exon boundaries [23, 24], CircMiMi offers users an optional function for checking whether the input BSJs are located at well-annotated exon boundaries and whether both the donor and acceptor splice junctions of a circRNA event are located at the annotated boundaries from the same annotated co-linear transcripts. For accuracy, this optional module also checks if the input BSJs of circRNAs are potential false-positives derived from ambiguous alignments with an alternative co-linear explanation or multiple hits. The exonic circle sequences flanking the BSJs (100 bp upstream and downstream sequences of the junctions; see Fig. 1b) are concatenated using bedtools [25]. The concatenated sequences are then BLAT-aligned [26] against the reference genome and well-annotated transcripts, with the default parameter set (-titleSize = 11 -stepSize = 11 -repMatch = 1024). A retained concatenated sequence should not map to an alternative co-linear matched sequence with > 80% similarity with the concatenated sequence or multiple hits with BLAT-mapping scores < 3 (Fig. 1b). Since different BLAT parameters may result in different alignment results, we realigned the concatenated sequences against the reference genome and well-annotated transcripts with a new BLAT-parameter set (-titleSize = 9 -stepSize = 9 -repMatch = 32,768) that is quite different from the default set. The alignment ambiguity checking is performed again. Only the concatenated sequences pass the alignment ambiguity checking based on both BLAT-parameter sets are retained. Such processes were demonstrated to effectively detect potentially false circRNA candidates from alignment ambiguity [18, 19, 27]. This module further provides a function to examine RCSs across the flanking sequences (RCS_across_) or within individual flanking sequences (RCS_within_) of the BSJs (see Fig. 1c). For examining RCS_across_ of a circRNA, both flanking sequences (± N nucleotides of the back-splice site; N is a parameter representing the number of nucleotides) are aligned each other using BLAST [28] with parameters –task blastn –word_size 11 –strand minus. For examining RCS_within_ of a circRNA, each individual flanking sequence is aligned itself using BLAST with the same parameters stated above. Of note, N is a user-defined parameter. The potential RCSs should be simultaneously satisfied the following rules: bitscore > 100, alignment length > 50 bp, and identity > 80%. The BLAST-parameters are set according to a previous study [29].

### Identification of circRNA-miRNA interactions

CircMiMi first generates putative exonic circle sequence for each circRNA event based on user-specified species, gene annotations and versions (Ensembl, Ensembl Metazoa, Ensembl Plants, or GENCODE) (Table 1). According to the mature miRNA sequences extracted from miRBase [30], two procedures were utilized to identify miRNA binding sites in the predicted circle sequences of circRNAs and construct potential circRNA-miRNA interactions. The first procedure screens potential miRNA binding sites at circRNAs using miRanda 3.3a (https://bioconda.github.io/recipes/miranda/README.html) [31]. Here we use a stringent parameter set with pairing score > 155 and energy score < − 20 recommended by a previous study [32]. For each predicted miRNA, the number of predicted binding site and the highest miRanda score of these binding site(s) were showed. The binding sites spanning the BSJ were also considered and represented. For human and mouse, CircMiMi provides the second procedure, which screens the miRanda-predicted miRNA binding sites and represents the binding sites supported by at least one Argonaute (Ago) CLIP-seq experiments. The Ago CLIP-seq data were downloaded from ENCORI [33] at http://starbase.sysu.edu.cn/. The liftOver tool [34] was employed to obtain the genomic coordinates of binding sites on the GRCh38 assembly.

**Table 1:** The species and the related annotations/resources used in this study

Species	Annotation/resource					circRNA-miRNA		miRNA-mRNA		
	E	G	EP	EM	mB	mR	ENC	mIB	mDB	ENC
*Arabidopsis thaliana*			V		V	V		V		
*Bombyx mori*				V	V	V		V		
*Bos taurus*	V				V	V		V		
*Caenorhabditis elegans*	V			V	V	V		V		
*Canis familiaris*	V				V	V		V	V	
*Danio rerio*	V				V	V		V		
*Drosophila melanogaster*	V				V	V		V		
*Gallus gallus*	V				V	V		V	V	
*Homo sapiens*	V	V			V	V	V	V	V	V
*Mus musculus*	V	V			V	V	V	V	V	V
*Oryza sativa*			V		V	V		V		
*Oryzias latipes*	V				V	V		V		
*Ovis aries*	V				V	V		V		
*Rattus norvegicus*	V				V	V		V	V	
*Sus scrofa*	V				V	V		V		
*Taeniopygia guttata*	V				V	V		V		
*Xenopus tropicallis*	V				V	V		V		

E, Ensembl; G, GENCODE; EP, Ensembl Plants; EM, Ensembl Metazoa; mB, miRbase; mR, miRanda; ENC, ENCORI; mTB, miRTarBase; mDB, miRDB

### Construction of circRNA-miRNA-mRNA interactions

After that, the miRNA-mRNA interactions were extracted from miRDB (version 6) [35] and miRTarBase (version 7.0) [36]. The former collected miRNA-mRNA axes predicted by MirTarget (version 4) [37] across five species; and the latter collected experimentally-supported miRNA-mRNA axes across 23 species. Of note, for the miRTarBase-collected miRNA-mRNA axes, we only considered the axes from the 18 species with Ensembl-based annotations (Table 1). For human and mouse, CircMiMi also extracted miRNA-mRNA interactions from ENCORI, in which the miRNA binding sites were predicted by one or more miRNA-binding prediction tools and supported by at least one Ago CLIP-seq experiments [33]. By integrating the circRNA-miRNA interactions with the miRNA-mRNA interactions, CircMiMi then generates circRNA-miRNA-mRNA interactions based on the common target miRNAs of the circRNAs and mRNAs.

For each input circRNA event, CircMiMi employs hypergeometric test to examine whether the identified circRNA-mRNA pairs are significantly co-regulated by miRNAs [38]. The statistical significance (*P* value) is determined as

$$P = \mathop \sum \limits_{i = s}^{{{\text{min}}\left( {t,c} \right)}} \frac{{\left( {\begin{array}{*{20}c} t \\ i \\ \end{array} } \right)\left( {\begin{array}{*{20}c} {N - t} \\ {c - i} \\ \end{array} } \right)}}{{\left( {\begin{array}{*{20}c} N \\ c \\ \end{array} } \right)}},$$

where *N* is the total number of miRNAs used to infer targets (circRNAs/mRNAs), *t* is the number of miRNAs that target the mRNA; *c* is the number of miRNAs that target the circRNA; and *s* is the number of miRNAs that target both the mRNA and circRNA. The *P* values are then adjusted across all circRNA-mRNA pairs using false positive rate (FDR) correction with Benjamini-Hochberg (BH) procedure [39]. A circRNA-miRNA-mRNA axis is retained if its circRNA-mRNA pair is significantly co-regulated by miRNAs at FDR < 0.05.

Finally, to visualize the identified circRNA-miRNA-mRNA regulatory networks on Cytoscape (https://cytoscape.org/) [40], the corresponding Cytoscape-executable file are provided. The NeuroCirc circRNAs and the corresponding information from other circRNA databases were downloaded from NeuroCirc at https://voineagulab.github.io/NeuroCirc/.

### Enrichment analysis

**Fig. 2:**
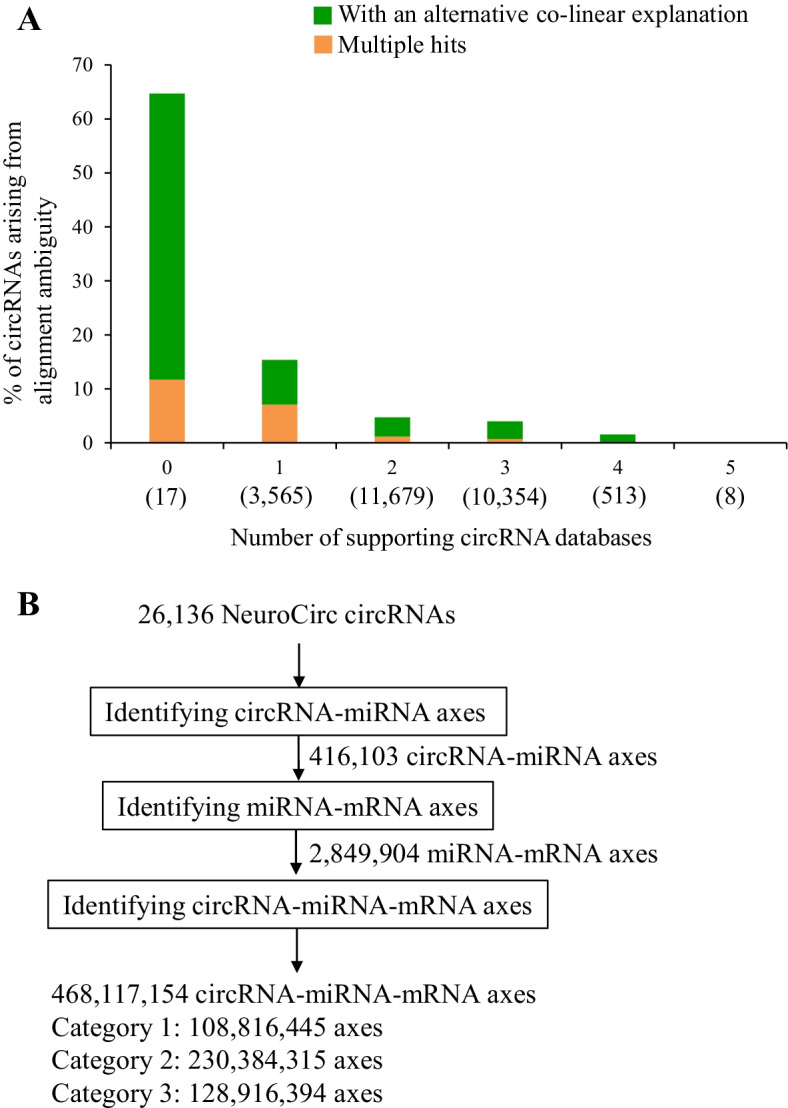
Identification of circRNA-miRNA-mRNA interactions based on the 26,136 circRNAs collected in NeuroCirc. **a** Alignment ambiguity checking for the NeuroCirc circRNAs. The NeuroCirc circRNAs are classified into six groups according to the number of supporting databases (0–5). The number of circRNAs for each group is shown in parentheses. **b** The flowchart of the CircMiMi process based on the NeuroCirc circRNAs

**Fig. 3:**
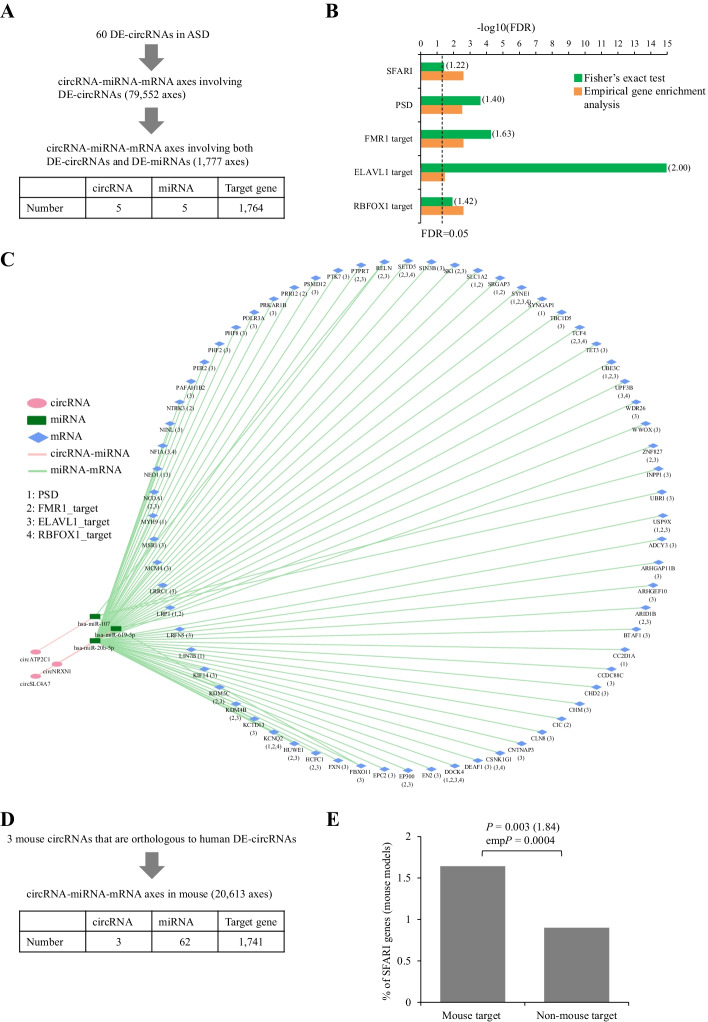
Cross-species functional analysis of ASD-associated circRNA-miRNA-mRNA regulatory axes. **a** The CircMiMi-identified circRNA-miRNA-mRNA axes that simultaneously comprised DE-circRNAs and DE-miRNAs in ASD. The DE-circRNAs [13] and DE-miRNAs [52] were previously identified using the RNA-seq data from the same postmortem brain samples. **b** Enrichment analysis of 5 groups of ASD-relevant genes for the target genes of the 1777 axes. ASD-relevant genes included ASD risk genes (i.e., SFARI genes), genes encoding PSD proteins, genes encoding transcripts bound by FMR1 (FMR1 target), ELAVL1 (ELAVL1 target), and RBFOX1 (RBFOX1 target). **c** Visualization of the 70 circRNA-miRNA-mRNA axes that simultaneously involved DE-circRNAs, DE-miRNAs, and SFARI genes according to the Cytoscape-executable file generated by CircMiMi. The SFARI genes that are also belonged to other groups of ASD-relevant genes are marked in parentheses. **d** CircMiMi-identified circRNA-miRNA-mRNA axes based on 3 mouse circRNAs that were orthologous to human DE-circRNAs. **e** Enrichment analysis of SFARI genes (mouse models) for the target genes of the axes illustrated in (**d**). For (**b, e**), *P* values were determined using one-tailed Fisher’s exact test and empirical enrichment analysis, respectively. *P* values were then FDR adjusted using Benjamini–Hochberg correction. The enrichment odd ratios were shown in parentheses

ASD risk genes (and genes from mouse models) were downloaded from the Simon Foundation Autism Research Institutive (SFARI) database (09-02-2021 release) at https://gene.sfari.org/ [41]. The lists of genes encoding postsynaptic density (PSD) proteins and targets of FMR1, RBFOX1, and ELAVL1 were downloaded from Lee et al.’s study [42]. We assessed each ASD-relevant gene list for the targets of the axes using the similar steps stated in our previous study [13]. For example, regarding the analysis of PSD gene enrichment for the target genes of the axes, we created a two-way contingency table with rows containing numbers of PSD and non-PSD genes and columns containing numbers of target genes and non-target genes. Here we used 20,070 protein-coding genes as the background set. We evaluated the statistical significance and odds ratio using one-tailed Fisher’s exact test with the *fisher.test* R function. *P* values were then FDR adjusted using BH correction. Human-mouse orthologous circRNAs were extracted from CircAtlas 2.0 [43] at http://159.226.67.237:8080/new/links.php. For empirical gene enrichment analysis [13] in Fig. 3b, e, we also took the analysis of PSD gene enrichment for the target genes of the axes in Fig. 2b as an example. We examined if the targets of the CircMiMi-identified axes (1764 genes; Fig. 3a) had a higher proportion (*p*_*obs*_) of the PSD genes compared to a null distribution of the proportion observed in the 10,000 times of random sampling. For each time, the same number (1764) of genes were randomly selected from the background set. The proportions (*p*_*i*_) in response to the 5 target groups for each ASD-relevant gene list were calculated. We then calculated the empirical *P* value (emp*P*) for each gene list as

$${\text{emp}}P = \frac{{1 + \mathop \sum \nolimits_{i = 1}^{10,000} {\text{number }}\;{\text{of}} \;(p_{i} > p_{obs} )}}{10,001}.$$

After that, emp*P* values were also FDR adjusted using BH correction.

### Implementation

The CircMiMi provides four main functions: (1) collecting all required resources; (2) checking BSJ, alignment ambiguity, and RCS; (3) identifying circRNA-miRNA-mRNA interactions; and (4) generating a Cytoscape-executable file (Fig. 1d). CircMiMi is implemented in Python 3 (tested with 3.6, 3.7, 3.8, and 3.9) and tested on major Linux distributions. CircMiMi is straightforward to install via “pip install circmimi”. The bedtools, BLAT, BLAST, and miRanda packages can be installed via “conda install -c bioconda bedtools = 2.29.0 blat blast miranda”. For user convenience, we also provide a program to automatically collect all required resources (genomic sequences, Ensembl- or Gencode-based annotation/version, miRBase, ENCORI, miRTarBase, and miRDB) in a specified folder via the command line “circmimi_tools genref” (Fig. 1d). If users do not specify the genome/annotation versions, CircMiMi automatically accesses the newest versions from the corresponding web sites. Moreover, the users can upload users-defined miRNA sequences or miRNA-target binding information into the specified folder (i.e., refs/) to determine circRNA-miRNA-mRNA interactions. The CircMiMi command lines also include the miRanda parameters for further screening circRNA-miRNA axes (Fig. 1d). The output tables include “summary_list” and “all_interactions”. The former sums up the results through the CircMiMi screening processes; and the latter represents all identified circRNA-miRNA-mRNA interactions.

## Result and discussion

We employed the circRNA candidates collected in NeuroCirc [22] as an example for analyzing circRNA-mediated interactions based on CircMiMi. Of note, NeuroCirc encompassed 26,136 circRNA candidates, providing an integrative view of circRNA expression in human brain tissues. After alignment ambiguity checking, we found that 1531 out of the 26,136 (5.9%) circRNA candidates were likely to be derived from ambiguous alignments with an alternative co-linear explanation (480 events) or multiple hits (1051 events) (Additional file 1: Table S1). Ambiguous alignments may originate from repetitive sequences or paralogous genes, which often result in false positive circRNAs [19, 27, 44]. Compared with five other well-known circRNA databases including circRNAdb [45], CircBase [46], CIRCpedia [47], CircFunBase [48], and CircAtlas [43], we can find that the percentages of circRNAs derived from alignment ambiguity remarkably decreased with increasing numbers of supporting circRNA databases, regardless of the types of alignment ambiguity (circRNA candidates with an alternative co-linear explanation or multiple hits; Fig. 2a). The percentage was significantly reduced from 65% (the circRNAs were detected in NeuroCirc only) to 0% (the circRNAs were detected in NeuroCirc and all the five databases examined) (Fig. 2a), supporting the association of circRNA reliability with alignment ambiguity. This result suggests that the circRNA candidates passing the alignment ambiguity checking may be relatively reliable for the further investigation.

Since back-splicing can be facilitated by RCSs residing in the sequences flanking circularized exons [3, 4, 20] and affected by the competition of RCSs across flanking regions (RCS_across_) or within individual regions (RCS_within_) [20], RCSs were often used to investigate circRNA formation (e.g., [49] and [29]). We found that the majority (76%; 19,865 out of the 26,136 circRNAs) of the NeuroCirc-identified circRNAs were observed to have RCSs (RCS_across_) in the flanking sequences of their back-splice sites (± 10 k nucleotides of the back-splice site) (Additional file 1: Table S1). Furthermore, 3847 out of the 19,865 circRNAs exhibited (RCS_across_ − RCS_within_) ≥ 1 (Additional file 1: Table S1). The RCS information may provide a starting point for further analysis of circularization, although the existence of RCS is not the absolutely necessary factor for circRNA formation in non-mammalian species [10] such as *Drosophila melanogaster* [50] and *Oryza sativa* [51]. Both the checks of alignment ambiguity and RCS are optional in the CircMiMi pipeline (Fig. 1d).

Regarding the 26,136 NeuroCirc circRNAs, we proceeded to construct potential circRNA-miRNA-mRNA interactions (Fig. 2b). We first identified potential circRNA-miRNA axes using miRanda and experimental data (Ago CLIP-seq data from ENCORI), respectively. As shown in Fig. 2b, a total of 416,103 circRNA-miRNA axes were identified. According to the miRNAs of the 416,103 circRNA-miRNA axes, we extracted miRNA-mRNA interactions from one database (miRDB) that contained bioinformatically predicted miRNA-mRNA axes and two databases (miRTarBase and ENCORI) that contained experimentally-supported miRNA-mRNA axes. A total of 2,849,904 miRNA-mRNA interactions were extracted. After that, the 468,117,154 potential circRNA-miRNA-mRNA interactions were constructed according to the common target miRNAs of the circRNAs and mRNAs. In terms of the experimental evidence of circRNA-miRNA axes and miRNA-mRNA axes, the identified circRNA-miRNA-mRNA interactions can be classified into three categories as follows (Fig. 2b).

*Category 1* 108,816,445 axes; both circRNA-miRNA axes and miRNA-mRNA axes were supported by experimental data.

*Category 2* 230,384,315 axes; either circRNA-miRNA axes or miRNA-mRNA axes was supported by experimental data.

*Category 3* 128,916,394 axes; other.

Since the circRNA candidates in NeuroCirc were derived from human brain tissue samples from neuronal differentiation datasets or individuals with neurodevelopmental diseases [22], it is of interest to investigate the circRNAs that were perturbed in neurodevelopmental diseases (e.g., Autism spectrum disorder (ASD) and schizophrenia) and the corresponding circRNA-miRNA-mRNA interactions. In terms of ASD, our previously identified DE-circRNAs (60 circRNAs) in ASD [13] were all included in NeuroCirc (Additional file 1: Table S1). On the basis of the 60 DE-circRNAs, CircMiMi identified 79,552 circRNA-miRNA-mRNA axes (Fig. 3a and Additional file 2: Table S2). Of the 79,552 axes, we further extracted 1777 circRNA-miRNA-mRNA axes that involved DE-circRNAs and DE-miRNAs simultaneously (Fig. 3a and Additional file 2: Table S2). Of note, the extracted DE-miRNAs [52] were derived from the same postmortem brain samples used for identification of the 60 DE-circRNAs. We then examined whether the target genes of the 1777 circRNA-miRNA-mRNA axes were implicated in ASD. We performed enrichment analyses (see Methods) for the gene sets previously implicated in ASD from SFARI [41] and other classes of ASD-relevant genes, including genes encoding postsynaptic density (PSD) proteins [53] and genes whose transcripts were bound by the three RNA binding proteins: FMR1 [54], RBFOX1 [55], and ELAVL1 [56]. Indeed, these target genes showed significant enrichment (all FDR < 0.05 by one-sided Fisher’s exact test and empirical gene enrichment analysis) for each class of ASD-relevant genes (Fig. 3b). The 70 circRNA-miRNA-mRNA axes that simultaneously involved DE-circRNAs, DE-miRNAs, and SFARI genes were illustrated in Fig. 3c (the detailed information of the identified interactions and ASD relevance were given in Additional file 2: Table S2). These data may provide a useful resource for further investigating regulatory mechanisms in ASD pathophysiology.

Moreover, considering the 5 DE-circRNAs examined above (Fig. 3a), 3 were orthologous to mouse circRNAs according to the CircAtlas annotation [43]. On the basis of the 3 mouse circRNAs, CircMiMi identified 20,613 circRNA-miRNA-mRNA axes, which were associated 62 miRNAs and 1741 target genes in mouse (Fig. 3d and Additional file 2: Table S2). Intriguingly, we found that these target genes were significantly enriched for the SFARI genes based on mouse models (Fig. 3e). This implies that the mouse circRNA-miRNA-mRNA axes derived from DE-circRNAs in human ASD brains is helpful for further investigation of regulatory mechanisms underlying ASD.

## Conclusion

In this work, we describe a well-tested stand-alone software called CircMiMi, which allows users to examine alignment ambiguity and RCSs of the input circRNA candidates, construct potential circRNA-miRNA-mRNA interactions, and visualize the identified circRNA-miRNA-mRNA axes. We utilized CircMiMi to identify circRNA-miRNA-mRNA interactions for all the circRNAs collected in NeuroCirc, a large-scale resource of circRNAs in the human brain. We further constructed circRNA-miRNA-mRNA interactions comprising DE-circRNAs and DE-miRNAs in human ASD and found that the targets of axes were enriched for ASD-relevant genes, providing important insights into the underlying molecular mechanisms in ASD etiology. Since CircMiMi can be applied to circRNA candidates derived from multiple species, we also constructed mouse circRNA-miRNA-mRNA interactions based on human-mouse orthologous circRNAs that were previously identified DE-circRNAs in human ASD brains. Our results revealed that the targets of such constructed axes were enriched for ASD risk genes based on mouse models. Taken together, this user-friendly tool may contribute to evaluation of circRNA reliability, investigation of circRNA formation, and cross-species functional analyses of circRNA-associated regulatory interactions, expanding our understanding of this important but understudied class of transcripts. CircMiMi will be continually updated as new experimental data of circRNA-miRNA and miRNA-mRNA interactions are available.

## Availability and requirements

Project name: CircMiMi.

Project home page: https://github.com/TreesLab/CircMiMi.

Operator system(s): Linux-like environment (Bio-Linux).

Programming language: Python 3.

License: MIT.

Any restrictions to use by non-academics: license needed.

## Supplementary Information

**Additional file 1: Table S1**. Alignment ambiguity and RCS checking for the NeuroCirc circRNAs. The file contains the results of alignment ambiguity and RCS checking for each NeuroCirc circRNA event.

**Additional file 2: Table S2**. The CircMiMi-identified circRNA-miRNA-mRNA axes. The supporting data for human-mouse orthologous circRNAs, circRNA-miRNA axes, miRNA-mRNA axes, and the relevance of the target genes to ASD were provided.

## Data Availability

The implementation of CircMiMi software package and source code are downloadable at https://github.com/TreesLab/CircMiMi. The web service is freely available at http://circmimi.genomics.sinica.edu.tw/. The CircMiMi-identified circRNA-miRNA-mRNA axes based on the 26,136 NeuroCirc circRNAs are available at http://treeslab1.genomics.sinica.edu.tw/CircMiMi/results/neurocirc/.

## Abbreviations

Ago: Argonaute
ASD: Autism spectrum disorder
circRNA: Circular RNA
CircMiMi: CircRNA-miRNA-mRNA interaction
DE: Differentially expressed
miRNA: MicroRNA
PSD: Postsynaptic density
RCS: Reverse complementary sequences
RCS_across_: RCSs across flanking regions
RCS_within_: RCSs within individual regions
SFARI: Simon Foundation Autism Research Institutive
